# Transitions from intensive eating disorder treatment settings: qualitative investigation of the experiences and needs of adults with anorexia nervosa and their carers

**DOI:** 10.1192/bjo.2022.535

**Published:** 2022-07-20

**Authors:** Danielle Clark Bryan, Pamela Macdonald, Valentina Cardi, Katie Rowlands, Suman Ambwani, Jon Arcelus, Eva-Maria Bonin, Sabine Landau, Ulrike Schmidt, Janet Treasure

**Affiliations:** Department of Psychological Medicine, Institute of Psychiatry, Psychology & Neuroscience, King's College London, UK; Department of General Psychology, University of Padova, Italy; Department of Psychology, Dickinson College, Pennsylvania, USA; Institute of Mental Health, University of Nottingham, UK; Care Policy and Evaluation Centre, Department of Health Policy, London School of Economics and Political Science, UK; Department of Biostatistics and Health Informatics, King's College London, UK; South London and Maudsley NHS Foundation Trust, Maudsley Hospital, UK

**Keywords:** Anorexia nervosa, carers, in-patient treatment, out-patient treatment, eating disorders

## Abstract

**Background:**

Relapse rates for individuals with anorexia nervosa after intensive hospital treatment (in-patient or full-time day care) are high. Better knowledge about the difficulties and opportunities that arise during this transition is needed to identify factors that support or hinder continued recovery upon discharge.

**Aims:**

The aim of this study was to explore the experiences of adult patients and their chosen carers on the process of transitioning from intensive eating disorder treatment settings to the community.

**Method:**

Semi-structured interviews were conducted with patients with anorexia nervosa (*n* = 11) discharged from day or in-patient care from specialised eating disorder units across the UK, and their chosen carers (*n* = 20). Data were analysed with inductive thematic analysis.

**Results:**

Four interrelated themes were identified for both groups. For patients, themes were continuity of care, ambivalence about continued recovery, the value of social support and a call for enhanced transition support. For carers, themes were the impact of the eating disorder on themselves and the family, perceptions of recovery and support post-discharge, the impact of previous treatment and care experiences, and desire to create a supportive transition process.

**Conclusions:**

The study provides an insight into the unique challenges that individuals with anorexia nervosa face upon leaving intensive treatment. A lack of post-discharge planning, support system and identity formation outside of anorexia nervosa were perceived as barriers to continued recovery. Patients and carers advocated for transition support that incorporates a phased, inclusive approach with accessible professional and social support in the community.

Anorexia nervosa is a severe, complex and life-threatening disorder.^1^ Definitions of recovery and relapse in anorexia nervosa vary, but relapse rates following discharge from eating disorder treatment are consistently high; a meta-analysis reported relapse rates of 31% at 1 year post-discharge,^2^ and a recent longitudinal investigation revealed that 40% of in-patient admissions required readmission, with 93% of these patients undergoing multiple hospital admissions within a short period.^3^ Readmissions are of particular concern given that a longer illness is associated with poorer clinical outcomes.^4^ National Institute for Health and Care Excellence guidelines recommend in-patient care as a second-line treatment for individuals with anorexia nervosa, reserved for those at high medical risk or risk of harm to themselves.^5^ In the short term, intensive eating disorder treatment settings are effective in improving eating behaviours and restoring weight,^6^ and provide a secure and accepting environment that facilitates peer and professional support.^7,8^ However, these potential benefits come with risks of reduced patient autonomy and individual responsibility in recovery once individuals return to the community.^9^ Two qualitative studies also revealed that psychological blocks to leaving in-patient treatment were increased by strong in-patient relationships, feelings of safety in hospital, concerns about loss of support and fear of relapse.^7,8^

Despite these concerns, few specific interventions to bridge the transition from intensive to less intensive forms of treatment for adult anorexia nervosa have been evidenced, with most of limited quality and replicability.^10^ Recent reports have called for this challenging issue to be addressed.^2,11^ Growing evidence suggests that carer support (e.g. from family members or partners) for adults with anorexia nervosa is beneficial for supporting and maintaining recovery post-discharge.^12,13^ However, high levels of burden and distress are reported by carers of individuals with eating disorders because of the emotional and physical demands of caregiving.^14^ There has been minimal consultation regarding patients’ and their families’ experiences of discharge to less intensive levels of care, and whether their needs are addressed. To expand current knowledge, this qualitative study explores the experience of transitioning back to the community from intensive care specialised eating disorder units across the UK, and the difficulties, opportunities and needs that arise during this period for people with anorexia nervosa and their carers.

## Method

The authors assert that all procedures contributing to this work comply with the ethical standards of the relevant national and institutional committees on human experimentation and with the Helsinki Declaration of 1975, as revised in 2008. All procedures involving human patients were approved by the London–Camberwell St Giles Research Ethics Committee (approval number 16/LO/1377). All participants provided written informed consent. This paper is reported in line with the Standards for Reporting Qualitative Research guidance.^15^

### Participants

Data for this study were collected as part of the qualitative evaluation of the Transition Care in Anorexia Nervosa through Guidance Online from Peer and Carer Expertise (TRIANGLE) trial (registered with the ISRCTN Registry under identifier ISRCTN14644379, on 8 December 2016), a multi-centre, randomised, controlled clinical trial investigating whether a shared intervention for patients and carers (ECHOMANTRA) in addition to treatment as usual (TAU) improves outcomes for people with anorexia nervosa after leaving intensive treatment.^16^ The study protocol provides a full description of the methodology of the trial.^16^ The inclusion criteria required patients to be aged 16 years or over, and admitted to a specialist eating disorder unit (in-patient or full-time day care) with a DSM-5 diagnosis of anorexia nervosa^17^ and a body mass index of ≤18.5 kg/m^2^ at admission. Patients nominated a carer (i.e. someone the patient defined as providing a close supportive relationship) and both required internet access. Patients were excluded if they were pregnant or had a severe chronic illness requiring treatment (e.g. diabetes mellitus, psychosis).

All participants who were randomised to TAU and had been in the TRIANGLE trial for at least 6 months were invited for interview. Recruitment for interview continued until data saturation was reached. Thirty-one participants (11 patients and 20 carers) participated overall, including eight dyads, i.e. patient and carer from the same family. The majority of carers interviewed were mothers of adult patients with anorexia nervosa who ranged in age from 17 to 33 years. Only one father was interviewed. The relationship of the remaining participants were heterosexual couples who tended to be older (age range 34–57 years) and consisted of three males who identified as carers and three female patients with anorexia nervosa. Demographic and clinical characteristics of the participants interviewed, including the index patients of carers interviewed, are shown in Table 1. All participants were interviewed individually. In total, patients and carers were recruited from 13 different specialist eating disorder units across the UK.

**Table 1:** Demographic and clinical characteristics of patients and carers interviewed

	Patients,^a^ *n* = 23 (proportion)	Carers, *n* = 20 (proportion)
Age, years, mean ± s.d.	24.78 ± 7.99	54.05 ± 8.15
Duration of illness, years, mean ± s.d.	8.35 ± 8.20	−
Number of months since last admission, months, mean ± s.d.	13.65 ± 3.56	−
Number of months since study baseline, months, mean ± s.d.	10.17 ± 2.61	9.85 ± 2.54
Body mass index at admission, mean ± s.d.	14.45 ± 2.21	−
Body mass index at study baseline, mean ± s.d.	16.77 ± 2.14	−
Gender, % female	20 (87.0%)	16 (80.0%)
Ethnicity, % White	23 (100%)	20 (100%)
Previously treated via		
Mental Health Act	9 (39.1%)	−
Community Treatment Order	3 (13%)	−
Living situation		
Living with parents(s)/relatives	15 (65.2%)	0 (0.0%)
Living with partner	3 (13.0%)	18 (90.0%)
Living in university accommodation	1 (4.35%)	0 (0.0%)
Living alone	4 (17.4%)	2 (10.0%)
Relationship within study dyad: ‘I am their … ’		
Child	20 (87.0%)	0 (0.0%)
Partner	3 (13.0%)	3 (15.0%)
Mother	0 (0.0%)	16 (80.0%)
Father	0 (0.0%)	1 (5.0%)

a. Characteristics are shown for patients interviewed (*n* = 11) and the index patients of carers interviewed (*n* = 12), to illustrate the full sample of experiences discussed in interviews.

### Data collection

Qualitative interviews were based on semi-structured topic guides (see Supplementary material available at https://doi.org/10.1192/bjo.2022.535) developed by authors D.C.B., P.M., K.R., V.C. and J.T. Researchers with lived experience of the illness (D.C.B. and K.R.) and with lived experience of caring (P.M.) drew upon experiential knowledge in addition to their academic expertise in the development and analysis of the interviews.

Participants were emailed to arrange a Skype call, conducted by an eating disorders researcher (P.M.), who was independent from the participant-facing TRIANGLE team. Interviews were audio-recorded using Skype for Windows Desktop, and transcribed verbatim. Personal identifying information was removed. All interviews were undertaken between April and May 2020. In light of the a full-scale COVID-19 lockdown imposed by the UK Government on 23 March 2020, additional questions were included to explore the initial impact of the pandemic on individuals with eating disorders and their carers. Data pertaining to COVID-19 were reported in a separate paper.^18^ Only data relating to experiences of transitioning from intensive treatment to the community before COVID-19 are reported in this study.

### Data analysis

Data were examined by thematic analysis with inductive coding.^19^ We took a critical realist approach, which focuses on a scientific method of data analysis while accepting that our understanding is inevitably constructed by our own experiences and perspectives.^20^ Two researchers (P.M. and D.C.B.) independently read the transcripts several times. ‘Utterances’ were coded, reviewed and refined, and an initial thematic framework was developed using the software NVivo for Mac (Release 1.0, QSR International Pty Ltd; https://www.qsrinternational.com/nvivo-qualitative-data-analysis-software/home). During six meetings, the researchers discussed emerging patterns until all transcripts had been analysed and data saturation reached. During the process, descriptive labels were altered to reflect the subject matter or deleted if deemed irrelevant to the research question. Initial analyses were reviewed among the wider research team, whereby the coding framework was discussed; themes and subthemes were then determined as coherently representing the complexity of the data.

## Results

### Patient themes

Four themes emerged from the patient data-set (see Table 2): continuity of care, ambivalence about continued recovery, the value of social support and a call for enhanced transition support.

**Table 2:** Frequencies of themes identified from patients’ experiences of the transition from intensive treatment for individuals with anorexia nervosa (*n* = 11)

	Patients,^a^ *n*	Utterances,^b^ frequency
Continuity of care		
Post-discharge treatment and care	11	45
Problematic transition and post-discharge care	6	35
Personally challenging aspects of the transition period	8	26
Ambivalence about continued recovery		
Self-efficacy, autonomy and motivation	11	46
Personal insights into the illness	6	31
Persistent challenges and obstacles	5	18
The value of social support		
Appreciation and acceptance of carer involvement	9	20
Views on support offered to carers	9	17
Empathy for the carer role	4	13
Benefits of peer support	4	10
Lack of carer support	3	10
A call for enhanced transition support		
Phased support and long-term planning	9	64
Inclusive support tailored to individual needs	5	17
Coordination and continuity between services	4	15

a. The number of patients who reported experiences that belong to that particular subtheme.

b. The total number of verbal statements that were made about that particular subtheme across all patient interviews.

#### Continuity of care

Patients gave accounts of the type of professional support they received following intensive treatment and highlighted how continuity of this care (or lack of it) affected their recovery. All described their experiences of post-discharge treatment and care, which varied from phased day care with specific treatment plans to less structured support.

‘I saw a therapist down at my local eating disorder service once a week [after discharge]. Being able to have access to a therapist at home was definitely helpful [and] I managed to get back into a routine’ (Patient 2).

Six patients described problematic transition and post-discharge care experiences. Typical issues included a perceived lack of communication and/or continuity between the services, premature/rushed discharge and little or no support offered.

‘We weren't given anything [after discharge] …  they said, “all right, we'll refer you to the community out-patient team” and put me back on a waiting list which is 2 years long’ (Patient 6).

Patients emphasised the personally challenging aspects of the transition period. A lack of structure and support, and increased responsibility for one's own well-being were particularly challenging. Concerns about managing weight and food intake were cited as frequent personal challenges.

‘They did the weight restoration …  but they didn't fix my mind so as soon as I was back home the old habits just kicked straight back in and now I'm finding it a struggle, a real, real struggle’ (Patient 3).

#### Ambivalence about continued recovery

This theme reflects the complex and often conflicting attitudes and feelings that patients experienced during the transition period. On the one hand, patients described increased motivation and autonomy in recovery, eloquently expressing insights into their illness and obstacles to recovery; but on the other hand, they described a continued struggle with change.

Across all interviews, patients demonstrated self-efficacy, autonomy and motivation. Several patients returned to work or began volunteering, whereas others practised mindfulness or other activities to maintain a sense of routine and purpose. The importance of nurturing social networks was also discussed as a motivating factor.

‘I don't like the fact I was put in hospital and I had to put my life on hold. I've got a lot of friends, I've got things I need to get done so the threat of if I have to go back [to hospital] …  then I would be set back even further’ (Patient 10).

Just over half of the patients evidenced personal insights into the illness that they had gained during intensive treatment and upon returning home.

‘It's harder now than it was when I was in the throes of anorexia. Back then I felt numb and powerful and now I feel vulnerable and angry and out of control, but I'm determined …  but I'll be totally honest, if I really need to be I can be very deceitful’ (Patient 6).

Approximately half the patients reported continued persistent challenges and obstacles regarding eating disorder-related thoughts and behaviours, including non-adherence with treatment, frustration at the enduring nature of an eating disorder and remaining in the precontemplation or contemplation phase of the illness.

‘I feel trapped between an absolute constant, constant fear of gaining, a constant desire for losing weight, a constant feeling that I'm too big and I want to get smaller’ (Patient 3).

#### The value of social support

Almost all of the patients interviewed expressed some appreciation and acceptance of carer involvement during the transition period. Examples given included being accompanied to appointments; help with meal planning, shopping and meal support; and a sense of being understood as an individual with an illness.

‘I definitely couldn't do it without my mum. I'm very fortunate in that she wants to be actively involved in my recovery’ (Patient 5).

Patients expressed specific views on support offered to carers. Although some felt that their carers had benefited from accessing information and support from services or carer support groups, others believed the psychoeducation provided was irrelevant to their own situation (particularly after multiple admissions or because of a severe and enduring form of eating disorder).

‘I think the effect on family is quite significant …  there was a parent support network at the hospital that I was in for in-patients, and they said that was quite helpful’ (Patient 1).

Several patients expressed understanding and empathy for the carer role, acknowledging the difficulties encountered in supporting them particularly in relation to deceptive behaviour. One patient discussed the challenges her partner faced in providing eating disorder support.

‘There's a fine line between them being your carer and your husband …  I'd love him to have somebody [to support him], probably a male, I feel like that would be easier for him’ (Patient 6).

However, three patients spoke candidly of a complete lack of carer support, citing unhelpful involvement perceived as detrimental to their well-being and recovery, or complete disengagement by family members.

‘Before I went into hospital, I decided to move out of my mum's house because she was very toxic, and she told me that I wasn't ill’ (Patient 10).

Several patients also spoke of the benefits of peer support, both for themselves and their carers, such as feeling emotionally supported and providing respite for the family. Types of peer support included informal links with other patients, organised peer groups and carer support networks.

‘I've got people who used to go to the service with me who I've got in contact with. We're struggling with the same kind of thing, so that's really helpful having that support’ (Patient 8).

#### A call for enhanced transition support

The majority of patients highlighted the importance and usefulness of a phased, supportive approach to transitions. This included the suggestion of a longer-term programme with the patient taking increasing responsibility for meal times and self-care to create a smoother transition home. Being able to maintain some contact with their treatment team when reducing intensive support was felt to create a more secure environment for patients to progress with their recovery.

‘I feel it's a process that you need to go through with a key worker or a therapist or whatever, just to walk you through it and make sure you're actually ok with the stepping-down process and the discharge process …  because it is such a big deal’ (Patient 11).

Half of the patients called for more inclusive support tailored to individual needs as opposed to a one-size-fits-all system. For example, an individual questioning their sexuality and gender identity and an individual with autism felt that they would have benefited greatly from a more personalised eating disorder treatment plan that addressed issues related to identity and comorbidities, and allowed appropriate support to be put in place at home before discharge.

‘They need a far bigger approach so that eventually people like myself can actually have a tailored approach …  because [otherwise] it has disastrous consequences for people like myself’ (Patient 4).

Finally, several patients advocated greater coordination and continuity between services. Suggestions included improved communication between treatment teams, with the aim of having better-informed staff, and continuity so that trust and therapeutic alliances built during their treatment could be nurtured.

‘I think there needs to be more accommodations between in-patient and out-patient treatment professionals consistently so that when you're discharged, it's not just on the patient to try and figure out what we need …  because I don't think we always know what we need’ (Patient 5).

### Carer themes

Four themes emerged from the carer data (see Table 3): the impact of the eating disorder on themselves and the family, perceptions of recovery and support post-discharge, the impact of previous treatment and care experiences, and desire to create a supportive transition process.

**Table 3:** Frequencies of themes identified from carers’ experiences of the transition from intensive treatment for individuals with anorexia nervosa (*n* = 20)

	Carers,^a^ *n*	Utterances,^b^ frequency
Impact of the eating disorder on themselves and the family		
Challenges to carers’ mental and physical health	19	106
Carers’ skills, strength and coping mechanisms	18	105
Impact on family relationships	10	22
Practical demands on, and challenges for, carers	9	11
Perceptions of recovery and support post-discharge		
Perceptions of inadequate post-discharge support received	17	119
Availability of post-discharge support	17	82
Transition challenges and perceived relapse	15	60
Perceived signs of improvement and patient efficacy	14	53
Impact of previous treatment and care experiences		
Positive experiences of care provided	15	58
Multiple admissions and non-adherence	13	33
Problematic in-patient care	10	58
Alternative treatment sought	8	25
Desire to create a supportive transition process		
Need for expert carer support and information	14	42
Inclusion in the recovery process	13	53
Social support and peer groups	13	41
Suggestions for improvements to the transition process	12	36
Need for holistic multidisciplinary approach to care	7	29

a. The number of participants who reported experiences that belong to that particular subtheme.

b. The total number of verbal statements that were made about that particular subtheme across all interviews.

#### Impact of the eating disorder on themselves and the family

Challenges to carers’ mental and physical health were discussed by nearly all carers as they reflected on what it felt like to live with uncertainty over the future of their loved one as well as experiencing isolation, blame, stigma, exhaustion, apathy, frustration, guilt, fear, anxiety and depression. Some carers reflected on their maladaptive coping mechanisms, such as overusing alcohol. Others expressed confusion about the nature of the illness and how to respond to it.

‘The effect really is quite catastrophic actually on my own physical and mental health. It's the first time I've ever had to approach a GP [general practitioner] with regards to my mental health’ (Carer 1).

Carers highlighted the skills, strength and coping mechanisms they used to address these challenges and bolster their strength to continue their role. Some carers sought out skills training programmes or psychoeducational books, or independently implemented adaptive cognitive mechanisms to cope with their situation. Participation in hobbies or their faith were described as acts of self-care. Several carers also engaged with their own therapist or consulted their general practitioner (GP). There were also several references to carers’ persistent, unfailing support, hope and resilience that built up in the face of adversity.

‘I just think it's really important to engage more …  we bought lots of books and we learned things through the carers support group meetings, and we read quite a lot … ’ (Carer 3).

Half of the sample spoke about the negative impact on relationships because of differing opinions on how to address the eating disorder within the family unit. Some partners spoke of their unique worries and the challenges faced in their relationships, and parents also highlighted concerns about the impact of anorexia nervosa on the well-being of siblings.

‘I try to be supportive but it does put a stress on a marriage …  we have different approaches and different ways of thinking. I've noticed that one of the things that happens is that you tend to blame each other as a way of coping …  which is wrong really’ (Carer 10).

The practical demands on, and challenges for, carers were referenced by nine carers. Carers experienced many logistical challenges, such as the timing or location of skills training courses, and struggling to arrange home leave with their loved one in a hospital far away. Some carers had given up work to care for their loved one, and others expressed guilt at having to juggle several responsibilities, such as childcare and work, alongside supporting their loved one.

‘ … trying to make sure whilst she was going out and about that she was eating properly. That was quite difficult to manage because I wasn't watching her 24/7, so I had to give her responsibility, but she wasn't really ready for it’ (Carer 7).

#### Perceptions of recovery and support post-discharge

This theme reflects the complex array of factors that carers perceived to have supported or inhibited continued recovery during the transition period. Seventeen carers conveyed perceptions of receiving inadequate post-discharge support, both for the patient and the family. Reasons included lack of resources, little or no treatment planning, and lack of coordination and continuity between the services. Carers perceived that this was inadequate for their loved one's continued recovery, and sometimes even harmful.

‘It's like none of the treatment happened at all and I think this is such a poor finish to it after all the work they've done with her …  not to have some continuity or concrete plan in place … because she's not had the support. The support's been awful’ (Carer 20).

Seventeen carers spoke about the availability of post-discharge support, including telephone calls from therapists or nurses in the context of out-patient/day care as well as support from other professionals, such as dieticians and local GPs. Carers gave several positive accounts of discharge planning having taken place when their loved one was still an in-patient, and of support offered to carers during the transition period.

‘I have a therapist from the eating disorder team who calls me once a week to offer support and talk through things, you know “How has the week been?”, which has been very helpful’ (Carer 11).

Most carers also referred to transition challenges and perceived relapse, citing concern around witnessing their loved one's deterioration in well-being. Carers reported noticing signs of eating disorder-related thoughts and behaviours, weight loss, negative moods and isolation that specifically occurred during the transition period.

‘She's gone back to being very snappy sometimes and she's gone back to being very in control of what everyone's eating in the house’ (Carer 14).

Nevertheless, more than half of the carers made a point of highlighting perceived signs of improvement in their loved one along with patient efficacy, e.g. engaging in activities, work, study or social life. There were also some reports of improved communication between carers and patients, which facilitated easier carer support and improved relationships.

‘For instance, today he decided that he was going to sit down and have a cup of tea and try and write something in a journal or do some Headspace [meditation app] or just try and relax for half an hour’ (Carer 12).

#### The impact of previous treatment and care experiences

Carers recounted experiences of care that were relevant to how their loved one managed post-discharge. These included positive experiences of care provided (e.g. GP care, early diagnosis and/or in-patient treatment) up to discharge deemed acceptable or useful to both patient and carer.

‘She had out-patient care every week with the psychiatrist …  there was lots of continuity there. It was actually fantastic, to be honest’ (Carer 19).

More than half of the sample reported experiences of multiple admissions and non-adherence that reflected harmful behaviours and multiple relapses, including refusal of treatment or self-discharge.

‘Well, she was in for 9 months at [in-patient unit], the previous year she was 9 months in [in-patient unit] and that sort of broke down as well … ’ (Carer 20).

Half of the carers perceived problematic in-patient care, such as disagreements with aspects of the care pathway or the legal framework for care of eating disorders, and challenging relationships with key team members.

‘Unfortunately, her experience in the residential unit was not a good experience and I feel very much that her mental health was compromised during that time although her physical health improved, her mental health deteriorated’ (Carer 3).

Several carers spoke of alternative treatment sought outside the mainstream National Health Service (NHS). These were either alternative therapies or private care, with one carer opting for specialist anorexia nervosa treatment outside the UK.

‘He liaised with the GP and then also with the non-eating disorder psychiatrist who was looking after him …  that made a massive improvement but that would not have happened if we hadn't tried to get private [help]’ (Carer 10).

#### Desire to create a supportive transition process

Carers provided considerable insight into their own needs and those of their loved one during the entire process, from intensive treatment through the transition period. Many expressed the need for expert carer support and information. This included improved signposting concerning where to obtain specialist information from service providers or support groups, and skills training for carers. The need for individually tailored guidance for male carers and patients was also emphasised.

‘As a male, you can be maybe a bit more direct or pointed, or a bit deeper pitched, so there's that perception that you're aggressive or angry …  I always felt there's not much out there for blokes’ (Carer 18).

Many of the responses reflected a desire for greater inclusion in the recovery process. Some carers felt excluded, either within their own family unit (e.g. one parent feeling less involved and helpful than another) or by the service and professionals.

‘She's a young adult and I know they have to be really careful about how they engage with families …  but she was not going anywhere other than coming home so I think it's really important to engage more with families to get them on board’ (Carer 3).

Over half of the carers interviewed reflected the positive benefits of social support and peer groups. There were several references to the usefulness of support from partners, other children, extended family or friends. Peer group support (in person or online) was also valued because carers could identify with others who encountered similar challenges.

‘I've got a couple of really good friends who kept checking in on me, the way you want the service to check in on you .… obviously I talk to my husband but you are living, breathing it together. You just want to offload it somewhere’ (Carer 8).

Over half of the sample gave suggestions for improving the transition process that were substantiated by aspects of care they felt they had lacked or had found helpful. Most suggestions included phased or tiered transition and a sense of continuity from in-patient treatment to home.

‘I think that there almost needs to be some step-down process …  there would be some support and they would be enabled to support themselves in terms of managing and cooking, all of that sort of thing’ (Carer 3).

Finally, seven carers mentioned the need for a more holistic multidisciplinary approach to care. Suggestions included therapies aimed at helping male patients, partners and fathers of people with eating disorders, and supporting individuals with comorbidities and issues related to identity.

‘So, you know, by holistic one means not only taking account of comorbidities, if we're going to call them that for the time being, but you know, other parallel problems, whatever it is … ’ (Carer 9).

## Discussion

We explored patients’ and carers’ perspectives and experiences of transitioning from intensive treatment for anorexia nervosa back to the community, with a focus on aspects that supported or hindered recovery during this period. All patients and carers described this period as challenging. The overarching themes were the need for continuity of care, the value of expert and social support, and the patient experience of ambivalence regarding sustaining progress toward recovery post-discharge.

In line with prior qualitative research, patients in this study expressed concerns about returning to anorexia nervosa thinking styles and behaviours when support levels were low and the next steps in their recovery unknown. The like-minded, empathetic community of intensive treatment settings, and the predictable structure of in-patient care, are widely reported as obstacles for individuals leaving anorexia nervosa treatment.^7,8,21^ Stepped day care settings and telephone check-ins by trusted members of patient's care team were cited as extremely valuable for building autonomy in recovery and increasing links to their home environments.

Ambivalence about change was illustrated through accounts of patients struggling to maintain recovery-focused behaviours once discharged back to the community. Conflicting feelings of shame and pride were expressed in recounting both successes and setbacks upon leaving treatment, which reflects the simultaneously positive and negative, ‘friend and foe’, associations that individuals have with anorexia nervosa.^22^ At the point of discharge, being confronted with whether to maintain the illness or recover from it amplifies the experience of ambivalence in recovery.^8^ An important finding in the study was that most patients, despite feelings of ambivalence, expressed a strong desire to continue to seek further help. However, patients were occasionally unable to access continued professional support (e.g. step-down or out-patient care) because of the lack of service provision, long waiting lists and geographical constraints.

Furthermore, many patients and carers perceived that they/their loved ones had been discharged from services solely because they had gained weight, despite this being contrary to clinician guidance.^5^ It was stressed that weight restoration without psychological support resulted in increased psychological distress, especially on the return home. Given that anorexia nervosa is commonly experienced as inextricable from the individual's sense of self, individuals experience (or fear experiencing) a distressing loss of identity through recovery.^7,23^

When consulted on areas of improvement in transition support, patients and carers believed that greater focus on the whole person in treatment (rather than only targeting anorexia nervosa) was critical for long-term recovery. This aligns with research that highlights that the process of building chosen and sustainable identities outside of the anorexic identity is a key therapeutic challenge required for successful recovery in the community.^23^ In our sample, there was a particular call to consider issues around comorbidities and gender and sexual identity. This reflects the high prevalence of significant comorbidities in patients with eating disorders,^24,25^ the high incidence of eating disorders in the LGBTQ+ population^26^ and the underrepresentation of males in peer-reviewed eating disorder research.^27^ The findings from this study suggest that wider issues related to identity need to be take into consideration via service-led interventions or appropriate community and family support, otherwise transitions back to the community are likely to re-encounter obstacles to recovery.

Social isolation and an eating disorder-focused identity are risk factors for maintaining and strengthening the illness.^21,22,28^ This may relate to this study's finding that meaningful engagement with peers and community life were major positive influences on recovery, and supported the formation of an identity outside of anorexia nervosa. Therefore, interventions that facilitate more engagement with peers, strengthen family support and increase social group membership in the community may be critical in promoting continued recovery after leaving intensive treatment.

### Strengths and limitations

To our knowledge, this is the first study that specifically explores both patients’ and carers’ experiences of transitioning from intensive treatment back to the community. Participants reported experiences from different clinical sites across the UK, which provide a great breadth of experiences for this study. However, the convenience sampling method may have biased recruitment. All feedback was provided by White, predominantly female participants, which reflects diagnosed anorexia nervosa prevalence rates.^29^ Our sample size was adequate for qualitative investigation, but the findings predominantly represent the views of mother–daughter relationships. The lack of diversity in our sample makes it hard to ascertain if our findings are generalisable to wider patient and carer experiences. A recent study has explored the perspective of partners and siblings in greater depth, and highlighted a vital need for additional guidance and support for these groups.^30^ However, from our sample, the appeal for more support tailored to individual needs and identities was evident, particularly for male patients, carers and LGBTQ+ individuals. We aim to widen access to this research by working with individuals in underrepresented eating disorder groups and from diverse cultural and socioeconomic backgrounds, with the aim of co-creating new tools that are relevant to a wider group of people.^31^

### Clinical implications

Transitions are essential in the management of anorexia nervosa. Moving back to the community from high-intensity care is particularly hazardous, with patients at increased risk of relapse and losing their treatment gains within the first months following discharge.^2,32^ The findings in this study support and inform the NHS's Long Term Plan, which aims to expand specialist community mental health services for adults with eating disorders, facilitating earlier access to care, as close to home as possible.^33^ This study found that improving access to psychological support to target inter- and intrapersonal aspects of the illness that are likely to be triggered upon returning to the home environment is crucial. Both patients and carers suggested that careful personalised planning with outreach support from eating disorder services may be valuable.

Digital interventions are increasingly facilitating communication with professionals, access to recovery resources and supportive communities for both patients and carers.^11,12,34^ People with lived experience may have an important bridging role by providing peer support.^35,36^

It is important, however, to be realistic about treatment expectations; recovery from an eating disorder involves the formation of a new identity, which usually takes time and might have a faltering course. The support of loved ones is invaluable. Fortunately, several relapse prevention trials and stepped-care models in adult services for anorexia nervosa are currently underway.^16,37,38^

## Data Availability

The data that support the findings of this study are available from the corresponding author, D.C.B., upon reasonable request. The data are not publicly available due to containing information that could compromise the privacy of research participants.

## Supplementary material

For supplementary material accompanying this paper visit http://doi.org/10.1192/bjo.2022.535. click here to view supplementary material
