# Supplementary material for: Transitions from intensive eating disorder treatment settings: qualitative investigation of the experiences and needs of adults with anorexia nervosa and their carers
Source: BJPsych Open. 2022 Jul 20;8(4):e137. doi: 10.1192/bjo.2022.535 (PMC9347315; doi:10.1192/bjo.2022.535)
Supplement: Supplementary file 1 [file S205647242200535Xsup001.docx]

**TRIANGLE PROJECT: SEMI-STRUCTURED TOPIC GUIDE**

**(TAU GROUP, PATIENTS)**

Interviewer introduces herself and purpose of interview – to explore the experience of the patient with regards to the overall transition process during eating disorder treatment.

“*We are interested in your point of view. Please tell us openly your experience of transitioning out of intensive treatment.”*

Specific points to address:

- No right or wrong answers
- Recording and confidentiality (only for transcribing purposes)
- Transcription – only small, anonymous quotes will be used
- Length of interview (up to 1hr)

1. After leaving intensive treatment, what support did you receive?

*Prompt:* Residential/daycare/out-patient?

1. Any support that was particularly helpful to you or your family?

*Prompt: Medical/nutritional support, peer groups*

1. What challenges, if any, did you face upon discharge?

*Prompt: what was unhelpful to you?*

1. How did you address these challenges?

*Prompt: What resources were available – support networks etc.*

1. How difficult or straightforward was it to keep up motivation to continue down the recovery path?

*Prompt: Any specific techniques used?*

1. To what extent have your carers been able to support you with the transition process?

*Prompt: Have they needed support? In an ideal world, what support would you like them to have?*

1. What suggestions do you have for a seamless transition process?
2. How have you found completing the TRIANGLE questionnaires so far?

Prompt: *What personally motivated you to complete the questionnaires? How did you feel after completing the questions*? Any general thoughts regarding your participation in the trial?

*“Thank you for your time today/this evening. This has been invaluable to us and to research into eating disorders.”*

**TRIANGLE PROJECT: SEMI-STRUCTURED TOPIC GUIDE**

**(TAU GROUP, CARERS)**

Interviewer introduces herself and purpose of interview – to explore the experience of the carer with regards to the overall transition process of their loved one with an eating disorder.

“*We are interested in your point of view. Please tell us openly your experience of caring for your loved one during the transition process out of intensive treatment.”*

Specific points to address:

- No right or wrong answers
- Recording and confidentiality (only for transcribing purposes)
- Transcription – only small, anonymous quotes will be used
- Length of interview (up to 1hr)

1. After leaving intensive treatment, what support did your loved one receive?

*Prompt:* Residential/daycare/out-patient?

1. After leaving intensive treatment, what support did you receive?

*Prompt:* family therapy? Skills training? Carer groups?

1. Any support that was particularly helpful to you or your family?

*Prompt: Medical/nutritional support, peer groups*

1. What challenges, if any, did you face upon discharge in supporting your loved one?

*Prompt: what was unhelpful to you? Helpful?*

1. How did you address these challenges?

*Prompt: What resources were available – support networks etc*

1. What was your experience with the transition process with regards to your own mental and physical wellbeing?

*Prompt: How did you take care of your own needs? How did these fit in with other demands of the caring role?*

1. What suggestions do you have for a seamless transition process?
2. How have you found completing the TRIANGLE questionnaires so far?

Prompt: *What personally motivated you to complete the questionnaires? How did you feel after completing the questions*? Any general thoughts regarding your participation in the trial?

*“Thank you for your time today/this evening. This has been invaluable to us and to research into eating disorders.”*
